# Rehabilitation and Return-to-Work of Patients Acquiring COVID-19 in the Workplace: A Study Protocol for an Observational Cohort Study

**DOI:** 10.3389/fresc.2021.754468

**Published:** 2022-01-31

**Authors:** Katrin Müller, Katharina Zwingmann, Tina Auerswald, Ivo Berger, Andreas Thomas, Anna-Lena Schultz, Eva Wilhelm, Rainer-Christian Weber, Franziska Kolb, Alois Wastlhuber, Sylvia Meder, Michael Stegbauer

**Affiliations:** ^1^Department of Social Science of Physical Activity and Health, Institute of Human Movement Science and Health, Faculty of Behavioural and Social Sciences, Chemnitz University of Technology, Chemnitz, Germany; ^2^BG Hospital for Occupational Disease Bad Reichenhall, Bad Reichenhall, Germany

**Keywords:** post-acute COVID-19 syndrome, occupational diseases, healthcare professionals, COVID-19^*^/rehabilitation, exercise capacity, cognition, psychological factors, return-to-work

## Abstract

**Background:**

In 2020, the novel coronavirus disease (COVID-19) developed into a worldwide pandemic. The course of COVID-19 is diverse, non-specific, and variable: Affected persons suffer from physical, cognitive, and psychological acute and long-term consequences. The symptoms influence everyday life activities, as well as work ability in the short or long-term. Healthcare professionals are considered particularly vulnerable to COVID-19 compared to the general population. In Germany, COVID-19 is recognized as an occupational disease or a work-related accident under certain conditions. Disease-specific rehabilitation is recommended for patients following acute COVID-19 to recover physical and neuropsychological performance and to improve work ability. Currently, there are limited findings on the short-term or long-term impact of COVID-19 as a recognized occupational disease or work-related accident, as well as on rehabilitation programs and associated influencing factors. Thus, the present research project will investigate these questions.

**Methods:**

For this observational cohort study, post-acute patients with COVID-19 as a recognized occupational disease or work-related accident according to the insurance regulations for COVID-19 will be recruited at the BG Hospital for Occupational Disease in Bad Reichenhall, Germany. All participants will complete a comprehensive multimodal and interdisciplinary inpatient rehabilitation program for a duration of at least 3 weeks, beginning after their acute COVID-19 infection and depending on their individual indication and severity of disease. Participants will complete medical, functional, motor, psychological, and cognitive measurements at four time points (at the beginning (T1) and end (T2) of inpatient rehabilitation; 6 (T3) and 12 (T4) months after the beginning of inpatient rehabilitation).

**Discussion:**

The present research project will help to assess and describe long-term effects of COVID-19 as a recognized occupational disease or work-related accident on physical and neuropsychological health, as well as on everyday activities and work ability of affected insured persons. In addition, this study will investigate influencing factors on severity and course of COVID-19. Furthermore, we will examine rehabilitation needs, measures, occurring specifics, and the feasibility of the rehabilitation procedure and disease development in the patients. The results of the intended study will further advance common recommendations for targeted and tailored rehabilitation management and participation in inpatient rehabilitation.

**Clinical Trial Registration:**

www.drks.de, identifier: DRKS00022928.

## Introduction

Since the beginning of 2020, the outbreak of the novel coronavirus disease (COVID-19: coronavirus disease 2019), caused by the severe acute respiratory syndrome coronavirus 2 (SARS-CoV-2), has developed into a worldwide pandemic, resulting in 199,466,211 confirmed cases and 4,244,211 deaths worldwide (World Health Organization/WHO; August 04, 2021) (1). According to the Robert Koch Institute (RKI), as of August 05, 2021, there were 3,780,985 confirmed COVID-19-cases and 91,730 persons have died from the disease in Germany (2).

Due to the rapid spread of COVID-19 and the lack of information on virus transmission, especially at the beginning of the pandemic, and the lack of hygiene concepts, healthcare professionals (e.g., physicians or nurses) have been the most vulnerable to COVID-19. According to the daily status report of the RKI for the coronavirus disease on June 25, 2021, 91,043 healthcare professionals have been reported as testing positive for COVID-19, 35,799 of whom work in hospitals and 2,030 of whom work in rehabilitation facilities. On that day, 3% of all infected healthcare professionals required hospitalization, no cases of death were registered and 90,800 patients were classified as recovered. A systematic review by Goméz-Ochoa et al. (3) showed that healthcare professionals show a higher prevalence of SARS-CoV-2 (18% of the 97 studies reviewed from 2020) compared to the general population. According to the RKI statements, it is presumable that the number of affected healthcare professionals will increase. According to the German Social Insurance Code (§9 SGB VII) (4) and the Ordinance on Occupational Diseases [Appendix 1 to the “Berufskrankheiten-Verordnung (BKV)”], COVID-19 can be recognized as an occupational disease 3101 when the insured person suffers from infections due to an activity warranting insurance protection. Therefore, to receive coverage, infection with SARS-CoV-2 must occur during the performance of the insured person's profession, e.g., in the field of (1) healthcare (e.g., hospitals, physicians' offices, pharmacies, nursing services), (2) social services (e.g., facilities for the care of children, youth, families, the elderly, and disabled persons, or persons suffering from mental illnesses as additional care), (3) laboratory work, or (4) activities with an increased risk of infection compared to (1) through (3). On the other hand, persons must be notably exposed to the danger of infection, suffer from at least marginal disease symptoms, and demonstrate intensive contact to the origin of the infection (“index person”) (5). According to the German Social Accident Insurance (DGUV, Department Statistics), 92,175 cases recognized with occupational disease 3101 were confirmed in Germany as of July 20, 2021. Furthermore, COVID-19 can be recognized as a work-related accident if infection with SARS-CoV-2 occurs because of the insured person's occupation without meeting the requirements for an occupational disease 3101. This requires being able to trace the infection back to the respective insured activity (e.g., employment, university/school, exercise of certain honorary positions). In this context, there must be intense and direct contact with an infectious person (“index person”) and the disease must manifest within 2 weeks of the last contact. Work-related accidents are terminated, externally influenced incidents that result in damage to health or death. Data from the DGUV (Department Statistics, July 20, 2021) confirmed 7,741 recognized cases of COVID-19 as work-related accidents. In the following, COVID-19 as an occupational disease or work-related accident will be referred to as Occupationally Acquired COVID-19 or OAC.

In general, the course of COVID-19 is diverse, non-specific, and variable, both in terms of presenting symptoms and severity of disease. According to the WHO, disease severity can be categorized into the following stages: mild (patients with symptoms but without evidence of viral pneumonia or hypoxia), moderate (with clinical signs of pneumonia), severe (with severe pneumonia and indication for oxygen therapy), and critical (e.g., with acute respiratory distress syndrome, thromboembolism, and/or multi-organ failure). Depending on disease progression, patients may require admission to a hospital or even an intensive care unit (ICU) with invasive mechanical ventilation. To date, the following predictors of a severe or critical course have been identified: Age (>50 years), male sex, dyspnea and persistence of fever, severe lymphocytopenia, and an increase of biomarkers (e.g., high sensitivity of C-reactive protein, proinflammatory cytokines), or underlying comorbidities (e.g., hypertension, heart diseases, diabetes, obesity, respiratory diseases, tumors, physical inactivity) (6–9). COVID-19 is also recognized as a multi-organ disease with the following extrapulmonary manifestations (10): neurological symptoms and diseases (e.g., dizziness, neuropsychiatric symptoms) or encephalopathy (11), cardiovascular symptoms and disease (e.g., myocardial damage, heart failure) (12, 13), renal, hepatic, and endocrine diseases (10), gastrointestinal symptoms, dermatological symptoms (10, 14).

According to the NICE (National Institute for Health and Care Excellence) guideline (15), there are clinical definitions for the initial illness and long-COVID at different times. *Acute COVID-19* is defined as signs and symptoms of the disease for up to 4 weeks. The guideline refers to *ongoing symptomatic COVID-19* when signs and symptoms last for a period of 4 up to 12 weeks. *Post-COVID-19 syndrome* is characterized as signs and symptoms for more than 12 weeks that cannot be explained by an alternative diagnosis and developed after an infection consistent with COVID-19. The term *long COVID-19* refers to continuing signs and symptoms following *acute COVID* and includes *ongoing symptomatic COVID-19* and *post-COVID syndrome*. At the beginning of the COVID-19 pandemic, little was known about long-lasting symptoms following acute COVID-19. The current state of research now indicates various physical, cognitive, and psychological symptoms in affected patients which persist over weeks or months and depend on disease severity (16–28). According to current publications, more than 1/3 of patients with *acute COVID-19* develop *long COVID-19* symptoms (23, 29–31). The following sections summarize current research on the effects of COVID-19 on physical, cognitive, and mental health, as well as work capacity.

Tuzun et al. (32) concluded that muscle weakness is quite common in hospitalized patients with COVID-19, especially in women with severe disease progression (32). This is supported by results from Paneroni et al. (33), who also found a high prevalence of impairment in muscle strength among hospitalized patients recovering from COVID-19 without previous locomotor disabilities (33). Other studies showed that COVID-19 patients suffered from acute impairments in physical function and capacity (25, 33–35). Aforementioned authors confirmed low (65%), intermediate (13%), and severe (10%) physical function impairments in patients with COVID-19 admitted to a sub-acute care unit (33). Belli et al. (34) demonstrated impairments in physical capacity and activities of daily living in COVID-19 patients in Italy after completion of acute treatment (34). Findings by Martin et al. (25) indicated that hospitalized COVID-19 patients had low functional exercise capacity at discharge and poor recovery after 3 months (25). Curci et al. (35) showed that post-acute COVID-19 patients had low physical capacity. Only 43.6% of the tested post-acute COVID-19 patients were able to walk and only few patients (18.8%) were able to perform the 6-Minute Walk Test (6MWT) with poor results. Furthermore, the authors' findings suggested that post-acute COVID-19 patients suffered from dyspnea and shortness of breath even during minimal activities, resulting in severe disability (35). Initial research results indicated that a COVID-19 infection can also have long-term consequences on physical function, strength, and physical capacity. Results by Bellan et al. (18) showed that 22.3% of tested COVID-19 patients had limited mobility 4 months after discharge (18). Results by Baricich et al. (16) supported this and found impaired physical performance in 32% of the tested COVID-19 patients at 3–6 months after hospital discharge (16). Findings by Blokland et al. (19) revealed that post-COVID-19 patients who were mechanically ventilated suffered from strongly deteriorated cardiorespiratory fitness and decreased peripheral muscle mass (19). Patients who recovered from mild or moderately severe COVID-19 can suffer from muscle weakness at least 12 weeks after diagnosis (28). There are currently few studies investigating the impact of COVID-19 infection on physical activity levels. However, current evidence suggests that physical activity level could decrease after a COVID-19 infection. Delbressine et al. (21) showed that weekly walking time decreased significantly after 3 months compared to pre-COVID-19. Although walking time increased after 6 months compared to 3 months after the onset of symptoms, walking time was still significantly lower compared to pre-COVID-19 (21). Among others, Tanriverdi et al. (28) investigated the physical activity level of recovered COVID-19 patients with mild or moderately severe diseases and showed that, even at least 12 weeks after diagnosis, physical activity level was low in 39.6%, moderate in 33.3%, and high in 27.1% of the participants (28).

Fatigue has evolved as a topic of major interest within the continuous findings of neurocognitive status post-COVID-19 infection. It is already common in other viral infections (e.g., infectious mononucleosis) (36) and may manifest as so-called chronic fatigue syndrome/myalgic encephalomyelitis in patients younger than 60 years with symptoms persisting for at least 6 months (ICD-10-GM-2021-G93.3). Six months after hospitalization, 63% of 1,655 Chinese COVID-19 patients reported fatigue and muscle weakness (23). A meta-analysis of 47,910 COVID-19 patients showed that 80% suffered from long-term symptoms, with 58% specifying fatigue as the first of five most common symptoms (24). In an international cohort study including 56 countries, fatigue ranked first among the most common symptoms reported after 6 months (20). The relevance of fatigue for everyday and workday activities is considered high, not least due to relapses triggered by exercise, physical/mental activity, and stress. Townsend et al. (37) examined predictors for COVID-19 fatigue symptoms and concluded that the occurrence of fatigue is independent from the severity of the initial COVID-19 infection. In addition, clustering of fatigue was noted in women and in cases with a prior diagnosis of depression/anxiety (37). Fatigue in long-COVID cases also appears to manifest together with several other neurological symptoms (20, 38). Common disorders include cognitive functions, such as concentration and memory, headache, muscle pain, continuous sense of smell and taste, or autonomic dysregulation (39). Within 80,000, mostly British, participants studied, infected individuals showed cognitive deficits in working memory, attention, and problem-solving abilities in comparison to persons without a COVID-19 infection (40). These objective deficits are consistent with subjective symptoms, such as reports of brain fog or anomia. Hospitalized, ventilated patients had a mean loss of 7 points on the Great British Intelligence Test, while non-hospitalized, non-treated individuals with confirmed COVID-19 infection also showed poorer cognitive performance compared to a healthy control group. Deficits “could not be explained by differences in age, education or other demographic and socioeconomic variables” [(40), p. 7]. Alemanno et al. (41) reported data from 87 sub-acute patients in an Italian COVID-19 rehabilitation program, 80% of whom had neuropsychological deficits confirmed by low scores on the Montreal Cognitive Assessment (MoCA) and the Mini-Mental-State Examination. They also indicated that cognitive function correlated with age, and that patients partially recovered 1 month after discharge from the hospital (41). Another study examining non-intensively treated COVID-19 patients 5 months after hospital discharge revealed processing speed deficits in 42.1%, delayed verbal recall deficits in 26.3%, and deficits in both processing speed and verbal memory in 21% of the cases (22). Further, there were also correlations found between several pulmonary variables (e.g., arterial oxygen partial pressure) and cognitive abnormalities. The authors concluded that the deficits examined have a major impact on work ability and everyday activities (22).

In addition to neurocognitive symptoms in patients with long-COVID, the results of a meta-analysis found that 45% of hospitalized patients showed symptoms of depression and 47% showed symptoms of anxiety (42). The frequency of depression and anxiety symptoms among COVID-19 patients decreased within several weeks after disease onset but was still present in 9% (26) to 13.6% (17) of patients for anxiety and in ~20% of patients for depression (26). Younger age, working in the healthcare sector, and female gender seem to be risk factors for depression and anxiety (43). Furthermore, 46.9% of ICU patients had symptoms of post-traumatic stress disorder (PTSD), and 23.5% of hospitalized patients had symptoms of PTSD 4–8 weeks after discharge. Obesity was associated with PTSD in the ICU group but not in the (normal) hospitalized group (44). In addition, the following risk factors for PTSD were mentioned: Age, female gender, healthcare professional, and perceived loss of control (27). Healthcare professionals, such as physicians and nurses, are considered particularly vulnerable for developing psychological symptoms after COVID-19 (45–47). Magnavita et al. (48) cited various reasons for the onset of psychological symptoms in COVID-19 infection, e.g., prolonged physiological and social deprivation due to social isolation and quarantine regulations, occupational experience of severe disease progression and mortality (also from colleagues), and persistent risk of infection and/or feelings of guilt from infecting relatives/friends (48).

The literature search resulted in very few studies investigating effects of COVID-19 on work capacity. In a meta-analysis including studies of patients infected with various corona viruses (SARS-CoV, MERS-CoV, SARS-CoV-2), Rogers et al. (27) showed that 446 of 580 patients from a total of 6 studies returned to work after an average of 35.3 months (27). Moreover, the expert group led by Kiekens et al. (49) referred to problems in performing work activities due to COVID-19 and recommended addressing this during follow-up treatment (49). Due to remaining symptoms up to 6 months after acute COVID-19, 45.2% of patients had to decrease working hours while 22.3% were unable to work (20). In the context of resuming work following COVID-19, it should be noted that working in the health care sector during the corona pandemic may have affected the mental health of staff. Lai et al. (50) examined 1,257 healthcare professionals caring for COVID-19 patients in China. These persons showed an increase in symptoms of depression (50.4%), anxiety (44.6%), insomnia (34.0%), and distress (71.5%) (50). Since stress might be a trigger for worsening symptoms, 85% of the patients reported improvements of symptoms with physical and mental training (20).

Taken together, there is a high prevalence of acute and even long-term effects of COVID-19 infection which affects physical, psychological, and cognitive health, as well as work ability. Healthcare professionals showed a particularly high prevalence of COVID-19 infection and are considered a vulnerable group for COVID-19 infection and its acute and long-term consequences. Based on these individual consequences, comprehensive and interdisciplinary rehabilitation of patients is required to recover from COVID-19 exposure and to reduce the long-term effects of COVID-19 on physical and mental health (49, 51). Due to the diversity of the disease progression, e.g., combined with invasive ventilation, complications after ICU treatment, effects on different organ systems, or comorbidities, the condition of post-acute patients at the beginning of rehabilitation varies widely and must be considered when planning and implementing personalized interventions (52). Based on the above-mentioned classification of disease severity, recommendations are made for outpatient (mild, moderate) or inpatient/intensive (severe, critical) care and further rehabilitative care needs (53). Depending on individual symptomatology, pulmonary, neurological, or cardiac inpatient or outpatient rehabilitation should be considered. The case management system of the German Social Accident Insurance Institution for the health and welfare services (“Berufsgenossenschaft für Gesundheitsdienst und Wohlfahrtspflege, BGW”) (54) additionally takes into account the incapacity to work during ongoing COVID-19 treatment. Several German hospitals provide a “Post-COVID-19-Check” (e.g., BG Hospital Berlin), meaning an interdisciplinary diagnostic procedure to examine the full disease symptomatology and derive individual therapy and rehabilitation concepts.

To date, only few studies have investigated the effects of rehabilitation programs after COVID-19 recovery. According to Belli et al. (34), primary rehabilitation measures after hospitalization revealed a significant positive effect on physical resilience and activities of daily living. Despite these effects, 53.3% of patients continued to show low physical resilience in the lowest range after rehabilitation. Therefore, subsequent measures to increase physical resilience are urgently needed (34). The retrospective analysis of data from cardiopulmonary rehabilitation of 28 COVID-19 patients after acute hospitalization also demonstrated improved physical capacity and subjective well-being after completing rehabilitation (55). The results of a randomized controlled trial by Liu et al. (56) with 72 COVID-19 patients confirmed significant improvements of respiratory parameters, 6MWT, health-related quality of life, and anxiety after 6 weeks of respiratory rehabilitation. However, there were no significant improvements of existing depressive symptoms (56). During the examination of inpatient rehabilitation of 100 COVID-19 patients after acute treatment, long-term outcome measures such as the Barthel Index or sit-to-stand-test correlated with the time spent in intensive care (57). The authors emphasized the usefulness of specialized rehabilitation units for severe COVID-19 infection, since greater improvements in some motor outcomes were found in patients who remained in intensive or prolonged acute care. Puchner et al. (58) also concluded that especially severe to critical COVID-19 patients with predominant needs for mechanical ventilation suffered from prolonged physical and mental limitations (e.g., muscle weakness, fatigue, neuropsychological problems) (58). Despite improvements in pulmonary function and physical performance status after an average of 24 days (± 5 days) of rehabilitation immediately after hospital discharge, some patients still showed limited diffusion capacity and neurological symptoms at the end of rehabilitation. This implies continued impairment for everyday activities or at work. Spielmanns et al. (59) compared the rehabilitation of patients with pulmonary disease with that of COVID-19 patients and found more pronounced impairment in COVID-19 patients with severe and critical courses of infection. Despite similar anthropometric values (e.g., mean age, days of pulmonary rehabilitation) and admission values, COVID-19 patients improved significantly more than pulmonary disease patients during rehabilitation in terms of physical performance and actual well-being (59).

In summary, rehabilitation programs can contribute to a reduction in physical, neurocognitive, and psychological restrictions, thereby stabilizing or even increasing patients' physical and mental performance after recovery from COVID-19. This is a prerequisite for maintaining the ability to work on a long-term basis after COVID-19, which is associated with economic costs. Considering the diverse and variable symptomatology, especially the associated physical limitations, social and occupational participation disorders are to be expected. However, there is currently no research focusing on the nature and extent of returning to work after COVID-19. Consequently, COVID-19 is associated with great challenges for the healthcare systems worldwide. In addition to acute patient treatment, long-term care of COVID-19 patients will be necessary after the acute phase due to illness-related impairments. However, based on the lack of longitudinal data and results, the sustainability of rehabilitation effects could not yet be confirmed. Indeed, since patients with severe to critical COVID-19 seem to benefit from rehabilitation soon after the acute phase of infection, rehabilitation programs are recommended for all hospitalized COVID-19 patients to avoid long-term adverse health outcomes (53, 60). However, inpatient rehabilitation programs should not only be offered directly after hospitalization and temporary discharge, but also weeks and months after the acute phase of COVID-19, depending on the existing symptoms of post-COVID (51). Together with the case control management of health services, rehabilitation should be provided to regain functionality in everyday and workday activities.

In this context, this study examines the following main research questions:

- What are the long-term outcomes of patients following OAC after undergoing an intensive rehabilitation program? Of particular interest are patients' physical capacity, psychological and cognitive well-being, and ability to work.
- Are age, gender, previous diseases, and employment at the time of exposure, as well as comorbidities associated with long-term outcomes of OAC of patients' physical capacity, psychological and cognitive well-being, and ability to work?
- Are physical capacity and psychological and/or cognitive well-being predictors for ability to work or return-to-work?

The results of the research questions and the documentation of the inpatient rehabilitation, will allow us to determine which special contents and features are feasible for sustainable rehabilitation management and occupational healthcare. Finally, in light of the current literature, we will derive recommendations when implementing inpatient rehabilitation with patients after acute COVID-19 at the BG Hospital for Occupational Diseases Bad Reichenhall.

## Methods and Analysis

### Study Design

**Figure 1:**
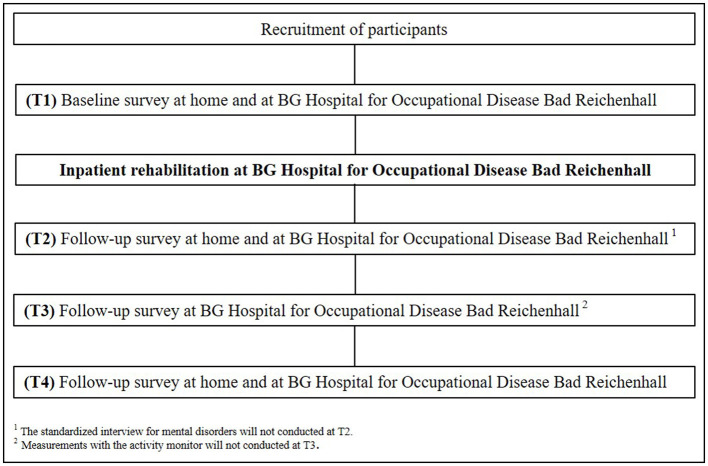
Study design.

The current study is an observational cohort study executed at the BG Hospital for Occupational Disease in Bad Reichenhall, Germany and at the Chemnitz University of Technology (TU Chemnitz), Germany. After successful screening for study eligibility, participants will complete medical, diagnostic, respiratory, neurocognitive, psychological, and physical performance measurements at four different time points (T1-T4). In addition to these, measurements using an activity sensor and a standardized interview for mental disorders will be performed at three different time points (see Figure 1). Data from the participants will be recorded over 21 months at the beginning (T1) and the end (T2) of inpatient rehabilitation. The follow-up time points 6 (T3) and 12 (T4) months after the beginning of inpatient rehabilitation consist of three examination days and will also be conducted by trained project staff at the BG Hospital for Occupational Disease Bad Reichenhall and at the TU Chemnitz.

### Post-COVID Rehabilitation Program

All subjects will participate in a comprehensive multimodal and interdisciplinary inpatient rehabilitation program at the BG Hospital for Occupational Disease Bad Reichenhall for at least 3 weeks after their acute SARS-CoV-2 infection, depending on patients‘ individual indication and severity of disease. The BG Hospital is specialized in treatment and holistic rehabilitation of occupational respiratory diseases and post-traumatic stress disorders, as well as work-related skin diseases. An interdisciplinary team of physicians, physiotherapists, sport therapists, psychologists, social professionals, nutritionists, nurses, and medical-laboratory assistants will implement the standardized rehabilitation program for patients after acute COVID-19. In addition to initial illness-specific diagnostics and continuous adaption of pharmacological treatments, the individual rehabilitation program will also include psychological therapy, sports therapy, physiotherapy, respiratory therapy, and health promotion interventions (e.g., smoke cessation, stress management, physical activity).

Psychological therapy, physiotherapy, and sports therapy are important components of the concept for post-COVID-19 rehabilitation at the BG Hospital. After initial psychological interviews with the patients at the beginning of the rehabilitation, and depending on the individual course of the disease, previous illnesses, and psychological processing strategies, the patients will participate in the following four education groups once a week, as recommended by a psychologist: “Anxiety”, “Depression”, “Sleep & Stress”, “Cognitive Training”. The cognitive training will be pre-discussed individually and will be carried out by the patients twice a week for 10–15 min using the computer-based program “Fresh Minder 2, 3, and 4” (Version 2.7.7, Version 3.5.5, Version 4.0.8, Ditzungen, Germany). “Fresh Minder 2” can help patients train their brain performance, memory functions, as well as their concentration in a playful and fun way. The “Fresh Minder 2” program offers different training modules to exercise a wide range of cognitive skills. The program covers acoustic and visual perception, working memory, sustained attention, flexibility, memory, divided attention, control of impulse, logical thinking, changing of perspective, spatial imagination, response capacity, computing, selective attention, speech comprehension, processing speed, visual spatial operations, visual scanning, visuo-constructive skills, visuo-motor skills, and word memory/word finding. Weekly group meetings for mindfulness, art therapy, and post-COVID-19 group therapy will be automatically scheduled for all patients. Furthermore, individual psychological and psychiatric sessions will be conducted if needed.

After assessing initial physical condition (cardiovascular und motor fitness), patients will receive an individual training program with sports therapy and physiotherapy. The program includes endurance training (e.g., Nordic walking, ergometer training), strength training (e.g., medical training therapy, stair climbing exercise), training of coordination, mobility and fascia training, as well as relaxation exercises (e.g., progressive muscles relaxation). Additionally, respiratory physiotherapy (e.g., mobility and respiratory techniques, inspiratory muscle training, specific manual therapy) will help to reduce breathing resistance and support patients in practicing breathing techniques, managing breathlessness, and training respiratory muscles. In general, the improvement of body awareness to recognize individual physical potential and limits of performance, as well as to improve self-management strategies is an important aim of sports therapy and physiotherapy.

### Participants

Based on the admission lists for rehabilitation after acute COVID-19 at the BG Hospital for Occupational Disease Bad Reichenhall, all patients concerned will be contacted by a trained study nurse from the clinic to inform them about the aims and the procedure of the current study in a standardized telephone interview. If patients agree to receive more information about the study, they will be sent written informed consent forms, the declaration of data protection, and the first assessments (T1) by mail.

Eligibility for study participation will be determined by the following inclusion criteria, regardless of age, gender, and migrant background: (1) COVID-19 as a recognized occupational disease or work-related accident, (2) patients in the post-acute phase of mild, moderate, severe, or critical COVID-19 as defined by the WHO, (3) no evidence of acute infectivity with SARS-CoV-2, (4) confirmation of coverage of costs for rehabilitation by the statutory accident insurers or the employers' liability insurance association responsible for the patient, and (5) voluntary participation. Participants will be excluded from the study if they were diagnosed with severe cardiovascular, respiratory, neurological, or musculoskeletal disorders, as well as mental (e.g., major depression) or neurocognitive (e.g., dementia) disorders prior to their infection with COVID-19.

### Outcome Measures

#### Disease Progression of COVID-19 and Comorbidities

A medical anamnesis by a physician will document the disease progression of COVID-19 (e.g., mild, moderate, severe, or critical course of disease), as well as 47 scientifically identified symptoms of post-COVID using a semi-standardized interview according to the current German COVID-19 and post-COVID guidelines (39, 61). Furthermore, previous diseases, individual diagnosed comorbidities, and the individual medication schedule (name, dosage, and frequency of intake for all prescribed medication) will also be recorded during medical anamnesis. Additionally, the questionnaires (see section Measurements to Assess Work Ability) require patients to state their current treatments during acute COVID-19 (e.g., hospitalization, ICU) at time point T1 and treatments for post-COVID-19 outside of inpatient rehabilitation (e.g., subsequent treatment by the general practitioner, ambulatory physiotherapy or psychotherapy) at time points T3 and T4.

#### Diagnostic Measurements

A routine laboratory blood withdrawal will be executed (7.5 ml whole blood) at all measurements. Analyzed blood parameters will include e.g., glucose, hemoglobin A1c, MBG, uric acid, GPT (ALAT), y-GT, alkaline phosphatase, creatinine (Jaffé), GFR (arithmetically following CKD-EPI), potassium (serum), NT-proNBP, calcium, cholesterol, triglyceride, TSH (basal), and leukocytes. Additionally, we will analyze TNF alpha, IL-6, IL-10, IL-17, since the inflammatory proceedings seem to have a high impact on the course of COVID-19 (62).

#### Respiratory Measurements

We will use spirometry, the most common pulmonary function test, to monitor lung health over time (63). All participants will perform body plethysmography (Masterscreen Body/diff, Vyaire medical, Höchberg, Germany) including single breath diffusion to measure several lung function parameters, e.g., forced expiratory volume in 1 s (FEV_1_), forced vital capacity (FVC), functional residual capacity (FRC), total lung capacity (TLC), and diffusion capacity of the lung for carbon monoxide (DLCO). While breathing ambient air, capillary blood gas samples (Cobas b 221, Roche Diagnostics intern., Rotkreuz, Switzerland) will be taken at rest, during the 6MWT, and spiroergometry to record partial oxygen pressure (PaO_2_) and partial carbon dioxide pressure (PaCO_2_). Additionally, we will conduct respiratory muscle testing according to Kabitz et al. (64) to quantify impaired respiratory muscle function with, e.g., maximum static pressure of inspiration (PI_max_) and respiratory capacity (P_0.1_/PI_max_) (64).

#### Neurocognitive Measurements

##### Montreal Cognitive Assessment

Cognitive performance will be assessed using the MoCA as the primary outcome in this area of the investigation. The MoCa is a short screening tool to identify cognitive impairments in patients, i.e., in memory, attention, or executive functions. Following the recommendations by Nasreddine et al. (65), participants who reach a MoCA score between 30 and 26 points will be classified as cognitively healthy, whereas individuals with a MoCA score of 25 or lower will be considered cognitively impaired (65). To avoid learning effects, different versions of the MoCA will be implemented at different measurements points: Version 1 at T1, Version 2 at T2, Version 3 at T3, and again Version 1 at T4.

##### Trail Making Test

The Trail Making Test will be conducted to assess attention deficits, executive dysfunction, and cognitive impairments. The paper-pencil neuropsychological test procedure consists of two parts. First, the participants have to connect numbers in an ascending order, then they have to connect numbers and letters in an alternating way (66). The time in seconds subjects require to complete the tasks will be used as the outcome measure. Depending on that measured time, the subject will be categorized into one of the following groups: “perfectly normal,” “normal,” “mildly impaired,” and “severely impaired” (67).

##### Digit Symbol Substitution Test

Used as part of the Wechsler Adult Intelligence Scale, the Digit Symbol Substitution Test is a neuropsychological test of sustained attention, visual spatial skills, response speed, and set shifting. In the paper-pencil task, participants have to write down the correct symbol associated with series of numbers from 1 to 9. The total of correct matches performed within 90 s is then counted. After the test, the correct number-symbol matches will be recorded and taken as memory count for the test (68).

#### Psychological Measurements

The self-administered questionnaire battery consists of validated and reliable instruments to detect psychological disorders for, e.g., somatoform disorders, depression, anxiety, post-traumatic stress disorders, as well as fatigue and coping strategies (see Table 1).

**Table 1:** Outcome measurements in the self-administered questionnaire.

**Outcome measurements**	**Instrument**
Origin, family status, household, education, employment and income, smoking, and drinking habits	Questionnaires of the German Health Interview and Examination Survey for Adults (“Studie zur Gesundheit Erwachsener in Deutschland,” DEGS) (69)
Socio-economic status	MacArthur Scale (German version) (70) Questionnaire for socio-economic status (DEGS) (71)
Activities of daily living	Baecke Questionnaire of Habitual Physical Activity (72)
Psychological well-being	Short interview for mental disorders—Open Access (Mini-DIPS-OA) (73) Patients Health Questionnaire (PHQ) (74) Hospital Anxiety and Depression Scale (HADS-D German version) (75) International Trauma Questionnaire (ITQ) (76) Somatic Symptom Disorder—B Criteria Scale (SSD-12) (77)
Fatigue	Brief Fatigue Inventory (BFI) (78) Fatigue Impact Scale (FIS) (79)
Coping	Brief COPE (80)
Quality of life	Short Form 12 (SF-12 Health Survey) (81)
Sleep quality	Insomnia Severity Index (ISI, German version) (82)
Work ability, employment	Work Ability Index (WAI, including comorbidities) (83) Brief Scale for Measuring Subjective Prognosis of Gainful Employment (SPE-Scale) (84) History of work ability and employment after acute COVID-19 (in comparison to DEGS) (71)
COVID-19 consequences on functional status	Post-COVID-19 Functional Status Scale (PCFS, German version) (85)
Post-COVID symptoms	Classification of symptom frequency [following (39)]

In addition, a psychologist will conduct a standardized interview for mental disorders with each patient at the beginning of rehabilitation (T1) and at time points T3 and T4.

#### Physical Performance Measurements

##### Spiroergometry

To assess participants' endurance capacity, the parameters oxygen intake, and ventilatory threshold will be measured using spiroergometry (Vyntus CPX, Vyaire medical, Höchberg, Germany). This is a diagnostic procedure in which increased physical strain within a ramp protocol is combined with the analysis of respiratory gases (86). The peak performance criterion is met when the participant reaches 70% of their anaerobic respiratory threshold. For this study, an “Ergoline” (Bitz, Germany) ergometer and the spiroergometry software “Masterscreen CPX” (Vyaire medical, Höchberg, Germany) will be used. During spiroergometry, an exercise electrocardiogram will be performed. Before spiroergometry, a resting electrocardiogram (Schiller AT 10 plus, Schiller Ag, Baar, Switzerland) will also be recorded.

##### 6-Minute Walk Test

The 6MWT, as the primary outcome in this area of the investigation, was chosen to assess functional exercise capacity. This test requires a walkway that is at least a 30m long. The walking course will be marked every 2 m. A cone will be placed on the ground to mark the turning points. The test coordinator will use a stopwatch to inform the participant when 6 min are over. Before starting the test, the participant has to sit on a chair close to the course for at least 10 min. The participant will be instructed to avoid talking while walking (87, 88). The outcome measure of the 6MWT is the distance in meters covered by the participant within 6 min. The 6MWT will be combined with a blood-gas analysis (Cobas b221, Roche Diagnostics intern., Rotkreuz, Switzerland) to provide information about the gas distribution of oxygen and carbon dioxide, the pH level, and the acid-base balance (89).

Finally, gait velocity of the first 10 meters of the 6MWT will be recorded to assess functional mobility, gait, and vestibular functions. The test coordinator will measure the time the participant requires to walk 10 meters, so that the velocity in seconds per meter can be calculated (90). Gait abnormalities will also be observed.

##### Handgrip and Leg Strength Test

We will use the digital grip dynamometer (JAMAR® Smart Hand Dynamometer, Performance Health Supply Inc, Cedarburg, USA) to assess handgrip strength. Participants sit upright on a chair without an armrest with their arm bent to 90°. Test duration is 3–5 s. Three trials are performed alternately with the right and left hand. The mean and highest values of the 3 trials for each hand will be analyzed (91). Measurements up to 90 kg will be included.

Additionally, we will measure the isometric maximal strength of quadriceps muscles using a functional press (Beinstemme v2, Schnell Trainingsgeräte GmbH, Peutnhausen, Germany) and the software aktivSYSTEM (aktivKONZEPTE AG, St. Ingbert, Germany). The measuring device will be set individually so that the sitting participant assumes the following joint angles for the measurement procedure: 100°(hip angle)/100°(knee angle)/90°(ankle angle). Isometric maximum force is tested against an insurmountable resistance (forcePOINT, aktivKONZEPTE AG, St. Ingbert, Germany). Three assessments will be executed with a rest of 15 s between each trial. The mean and highest value of 3 trials will be analyzed.

The 1-Minute-Sit-to-Stand-Test will be used to measure participants' functional performance. For this test, participants sit on a chair with a seat height of 46 cm without armrest. Participants are instructed to stand up and sit down as often as they can within 1 min. It is important that the knees are not bent while standing and that the participants do not actively use their arms. The test coordinator counts the completed repetitions during 1 min (92).

##### Fine Motor Task

The Purdue Pegboard Test [(93); Model 32020A, Lafayette Instrument] will be used to test participants' manual dexterity, fine motor performance, and cognitive abilities. The pegboard consists of two parallel rows of 25 holes each. Four cups are located at the top of the board containing pins (left and right), washers (middle left), and collars (middle right). During first test battery, the dominant hand places pins into the holes in concomitant order from the top down in 30 s. After that, the non-dominant hand is tested using the same assignment (second test battery). During the third test battery, both hands are used to simultaneously place pins in the holes within 30 s. Finally, pins, washers, and collars are placed into the holes in a certain order (1. right hand: pin, 2. left hand: washer onto pin, 3. right hand: collar onto washer, 4. left hand: washer onto collar) with both hands working together complementarily. Participants have 1 min to complete this fourth test battery. Instructions include pegging as many elements as possible within the given time during each test battery and not picking up a dropped element, but returning it to the cups for a new element. One-trial administration of the Purdue Pegboard test will be executed and number of elements will be counted for each test battery.

##### Balance Tests

The first and third blocks of the GGT-Reha balance test (“Gleichgewichtstest-Rehabilitation”) (94) will be used to measure motor balance. A physiotherapist or sports therapist will conduct the balance test at the beginning of therapy to determine the success of the subsequent rehabilitation period. Each block consists of six exercises (e.g., stand on an unstable surface with feet hip-width apart and closed eyes) that are performed in an increasing order of difficulty. If the participant cannot complete two consecutive tasks, the subsequent tasks of that block are not performed. The balance test will be conducted in a gymnasium, and an unstable base as well as a stopwatch will be necessary. The patient's task is to hold the position for 15 s. The therapist rates the subject's performance on a scale from 0 to 3 (0 = did not fulfill the task, 3 = fulfilled the task perfectly) and reports the amount of time in seconds each exercise position was maintained (if the patient is not able to hold the position for the requested 15 s) (94).

##### Physical Activity and Sleep Behavior

Physical activity and sleep levels will be measured using objective and subjective instruments. An activity monitor (ActigraphGT9xlink®, Actigraph, Pensacola, FL, USA) will be used to objectively assess physical activity. This three-axial accelerometer is the gold standard of activity monitors for patients with chronic diseases, e.g., chronic obstructive pulmonary disease (COPD) or diabetes, and is recommended as a valid tool to measure physical activity. The activity monitor will be placed on the right side of the waist and should be removed for swimming or showering. Participants will wear the monitor on 7 consecutive days over 24 h at time points T1, T2, and T4 at home. Additionally, participants will receive a diary in which they should record sleep and times when they did not wear the activity monitor. Data will be analyzed using the ActiLife 6 Software (Actigraph, Pensacola, FL, USA). Data from participants who wear the activity monitor <4 days and data from days with <480 min wear-time will be excluded. Wear and non-wear times will be analyzed using methods described by Choi et al. (95). We will use the floating window algorithm and 20 min of consecutive zero counts to identify the non-wear time. To determine physical activity levels, we will select the classification of physical activity intensity according to Freedson et al. (96), and classify <100 counts per minute (cpm) as “Sedentary”, 100–759 cpm as “Light”, 760–5,720 cpm as “Lifestyle”, 5,725–9,498 cpm as “Vigorous” physical activity behavior. Sleep duration will be calculated based on sleep onset and offset times reported in the sleep diary. The following variables will be analyzed for sleep: sleep onset (the first minute that the algorithm scores “asleep”), total sleep time (the total number of minutes scored as “asleep”), sleep efficiency (number of sleep minutes divided by the total number of minutes the patient was in bed), wake after sleep onset (the total number of minutes the subject was awake after sleep onset occurred), average awakening (the average length in minutes of all awakening episodes), and total counts (actigraphy totals summed for the entire sleep period). Additionally, participants will complete the 7-item Insomnia Severity Index (82) assessing the severity and impact of insomnia. Furthermore, the Baecke Physical Activity Questionnaire (72) will be used. This is a self-administered, 16-item questionnaire within 3 categories of occupational activities, sport activities, and recreational (leisure) activities.

#### Measurements to Assess Work Ability

Employment status and ability to work will be assessed using the following questionnaires: the Work Ability Index [WAI; (83)] as primary outcome in this area of the investigation, the Brief Scale for Measuring Subjective Prognosis of Gainful Employment [SPE-Scale; (84)], and the History of work ability and employment after acute COVID-19 [in comparison to DEGS; (71)] (see also Table 1). These questionnaires assess functional recovery in terms of work ability and employment (e.g., days from diagnosis to first partial/complete return to previous work, work interruptions due to sick leave) for the subsequent period up to 12 months after the start of inpatient rehabilitation. Additionally, physicians will record detailed information during medical anamnesis.

#### Questionnaire Battery

The participants will complete a questionnaire battery at all measurements. The questionnaire battery will include the following outcome parameters: socio-economic status, quality of life, work ability and employment. The self-administered questionnaire includes validated instruments and self-generated items (see Table 1).

#### Documentation of the Rehabilitation Process

To collect data about the general rehabilitation process and to draw conclusions for future rehabilitation management, all rehabilitation staff must document feasibility aspects and particularities in the implementation of inpatient rehabilitation, as well as critical events that might occur during individual inpatient rehabilitation for each patient. Furthermore, the rehabilitation staff involved in the study will take part in a standardized interview on these topics. Documentation of the inpatient rehabilitation process will also be supplemented by recording the subjective individual physical and psychological well-being (scale from 0 = very bad/not present to 10 = very well/strongly pronounced) using patient questionnaires before and after rehabilitation. Based on these results, comparisons can be made with the results of the other objective measurements. To document subjective functional recovery during the follow-up period after inpatient rehabilitation, subjective individual physical and psychological well-being will also be measured at T3 and T4. In addition, at measurement time points T1, T3, and T4, patients will be asked by questionnaire [compared to DEGS; (71)] to indicate post-COVID-19 treatment outside of inpatient rehabilitation (e.g., follow-up treatment by the general practitioner, ambulatory physiotherapy, or psychotherapy). Results of the assessments will be summarized in the reports from physicians and psychiatrists at BG Hospital to coordinate further treatment.

### Data Management

#### Data Collection and Management

Participant information will be recorded using an individual identification code. All hard copy forms will be stored at TU Chemnitz and BG Hospital in locked cabinets accessible only by project staff. Electronic data will be stored on a secured password-protected computer. The databases will not contain subject identifiers. The data linking subject identifiers and the individual identification codes will be stored separately. Data quality will be promoted by double data entry and range checks for data values. Only project staff will have access to the final trial dataset.

#### Data Monitoring

As no adverse events are expected, no data monitoring committee will be established to be responsible for data monitoring, interim analyses, and auditing. Study participants will be monitored at all times by trained project staff who will intervene if adverse reactions are observed during measurements.

### Sample Size Calculation

The sample size is calculated using G^*^Power 3.1.9.4 (University of Kiel) for the expected results of physical performance (6MWT) and mental well-being (depression). While 6MWT is a standardized measurement to assess physical capacity in patients with chronic disease during rehabilitation, e.g., COPD, (87–89) it was used as the primary outcome of physical performance for sample size calculation. 6MWT was also conducted in the first inpatient rehabilitation studies with patients with COVID-19 and showed good feasibility (31, 55). Multiple regression analyses assess the baseline situation of patients after acute COVID-19 with regard to physical resilience or mental health at the beginning of the inpatient rehabilitation. Due to the novelty of the disease, there are no representative comparative values for associations between COVID-19 and physical resilience and mental health. Therefore, the calculation of sample size in the cross-sectional analysis refers to the generic effect size measure *f*^2^. According to Cohen (97), a mean effect size of *f*^2^ = 0.15 will be used to calculate the sample size. Results from studies on correlations in patients with pneumological diseases (e.g., COPD) are used to justify this selected effect size in more detail. The study by Zeng et al. (98) identified age, severity of disease, and quality of life as significant predictors for physical performance measured by 6-minute-walking-distance in patients with COPD with a medium effect size of *f*^2^ = 0.21. In a regression model, von Leupoldt et al. (99) showed that depression is one out of 6 predictors (e.g., age, gender, forced expiratory volume in 1 s) for the completed 6MWT in COPD-patients in outpatient rehabilitation with a small effect size of *f*^2^ = 0.06 (71). Due to the heterogeneity of study results, a medium effect size of *f*^2^ = 0.15 can be assumed for the calculation of sample size to present associations between physical performance and mental health. With a planned power of 80% and significance level of 5%, 92 data sets are required, considering up to 5 potential predictors (e.g., pulmonary function values, blood gas values, age).

To detect the outcomes over time, differences between 2 dependent samples (T1 and T2) will be analyzed. The minimal clinically important difference between two measurement time points is 30 meters for the 6-minute-walking-distance (88). According to experience from our own studies on physical performance in patients with occupational respiratory diseases, standard deviations between 80 and 100 m are to be expected (100, 101). Assuming a standard deviation of 100 m (due to heterogeneity of the sample) and an expected effect size of d = 0.3, 71 data sets are required with a power of 80% and significance level of 5%.

While a drop-out rate of 25% is expected during the study period of 21 months, a total sample size of at least 115 patients will be included.

### Statistical Analysis

IBM SPSS Statistics Version 28 and AMOS Version 28 (IBM Corp., Armork, NY, USA) will be used for all statistical analyses.

First, to determine the existing outcomes of COVID-19 on physical capacity, psychological and cognitive well-being, and the ability to work at the beginning of inpatient rehabilitation (T1), the population will be descriptively characterized by all measured outcomes. Data from participants at the end of inpatient rehabilitation (T2) and at the follow-up time points (T3, T4) will be analyzed to further evaluate the long-term outcomes of patients with COVID-19 undergoing an intensive rehabilitation in terms of physical capacity, psychological and cognitive well-being, and the ability to work. Normal distribution and homoscedasticity of the sample will be realized using the paired *T*-test or Wilcoxon-test. Depending on the feasibility for the current population, group differences (e.g., age, gender, pre-existing conditions, comorbidities, current employment) in the long-term effects of COVID-19 on physical capacity, psychological and cognitive well-being, and the ability to work will be analyzed separately for the different time points (T1, T2, T3, T4) using different statistical methods as follows. Taking into account the number and size of groups, normal distribution and homoscedasticity of the sample, parametric or non-parametric statistical methods such as the *T*-test, Mann-Whitney *U*-test, ANOVA, or the Kruskal Wallis test will be used. To evaluate the associations between the different outcome measures of physical capacity, psychological and cognitive well-being, and ability to work, correlation analysis will be performed to measure the strength of the relationship between two variables, e.g., 6MGT and HADS-D or 6MGT and WAI. Depending on these results and current insights from the literature, multivariate and logistic regression analyses accounting for confounders such as age, gender, or comorbidities will be conducted to identify predictors or meditators of the long-term outcomes of COVID-19. For example, a logistic regression analysis will be used to examine the relationship between physical capacity, psychological and cognitive well-being and the dependent variable return to work 6 or 12 months after the start of inpatient rehabilitation. For all analyses, Bonferroni correction for multiple testing will be applied and statistical significance level will be set to *p* < 0.05. After inspecting the amount and pattern of missing data and outlier values, the most appropriate procedure for addressing both factors will be selected.

## Conclusion

Using the planned study protocol, long-term effects of OAC on physical capacity, mental health, and work ability will be assessed and described in detail. With a follow-up period of 12 months after the start of inpatient rehabilitation, the project makes an important contribution to the state of knowledge about the long-term effects of the disease. In addition, this study will present associations between physical capacity, mental health, and work ability, as well as the influence of age, gender, comorbidities, and occupation. Moreover, measures, occurring peculiarities, and the feasibility of the inpatient treatment procedure for patients with OAC will be documented. During and at the end of the project period, (interim) results and findings will be summarized in information brochures and presented at information events for the accident insurance institutions. When interpreting the results, it should be considered that the target group is a selective sample of patients with the diagnosis of COVID-19 as a recognized occupational disease or work-related accident. In Germany, the BG Hospital for Occupational Disease Bad Reichenhall is a clinic specialized in the treatment and rehabilitation of occupational diseases and consequently appropriate for the investigation of this sample group. A randomized, controlled study design cannot be applied in the context of the current research project. Since the progression of COVID-19 is acute and associated with direct health-related consequences, it is ethically and medically irresponsible to deny affected insured persons inpatient rehabilitation over a longer period. Nonetheless, the results of the current study will contribute to further developing recommendations for monitoring and coordinating targeted and tailored inpatient rehabilitation in the context of sustainable rehabilitation management for patients after COVID-19. This can help optimize the procedures and processes of post-COVID rehabilitation with the aim to improve the patients‘ physical and mental health, and to support individual occupational ability. Finally, the results will provide a basis for further research, such as the development of disease-specific, health-promoting, and rehabilitative strategies for the sustainable healthcare of patients with COVID-19.

## Ethics Statement

The studies involving human participants were reviewed and approved by the Ethics Committee of the Chemnitz University of Technology (TU Chemnitz), Faculty of Behavioural and Social Sciences (number V-427-17-KM-COVID-19-18022021) and by the Ethics Committee of the Bavarian State Medical Association (number 21092). The patients/participants provided their written informed consent to participate in this study.

## Author Contributions

KM: conceptualization, methodology, investigation, writing—original draft, visualization, supervision, project administration, and funding acquisition. KaZ: methodology, investigation, writing—original draft, and editing. TA: investigation, writing—original draft, and editing. AT, IB, A-LS, EW, R-CW, FK, AW, and SM: investigation and writing review and editing. MS: conceptualization, investigation, writing review and editing, and supervision. All authors contributed to the article and approved the submitted version.

## Funding

This research is funded by the German Social Accident Insurance (Deutsche Gesetzliche Unfallversicherung e.V., DGUV) [grant number: FF-FB 0326]. This funding source had no role in the study design, methods, or in writing the report. The publication of this article was funded by Chemnitz University of Technology.

## Conflict of Interest

The authors declare that the research was conducted in the absence of any commercial or financial relationships that could be construed as a potential conflict of interest.

## Publisher's Note

All claims expressed in this article are solely those of the authors and do not necessarily represent those of their affiliated organizations, or those of the publisher, the editors and the reviewers. Any product that may be evaluated in this article, or claim that may be made by its manufacturer, is not guaranteed or endorsed by the publisher.

## Abbreviations

6MWT: 6-Minute Walk Test
BG: “Berufsgenossenschaft&#8221
= German Social Accident Insurance Institution
COPD: Chronic Obstructive Pulmonary Disease
COVID-19: coronavirus disease 19
cpm: counts per minute
DGUV: “Deutsche gesetzliche Unfallversicherung&#8221
= German Social Accident Insurance
ICU: intensive care unit
MoCA: Montreal Cognitive Assessment
OAC: Occupationally Acquired COVID-19
PTSD: posttraumatic stress disorder
RKI: Robert Koch Institute
SARS-CoV-2: severe acute respiratory syndrome coronavirus type 2
T1/T2/T3/T4: measurement time point 1/2/3/4
TU Chemnitz: Chemnitz University of Technology
WHO: World Health Organization.
